# Diseases of the Lungs

**Published:** 1896-01-18

**Authors:** 


### Progress in Medicine.

### DISEASES OF THE LUNGS AND PLEURÆ.

(*Continued from p.* 232).

**Tuberculin.**—Favourable results from the employment of Koch's tuberculin are reported by C. H. Denison,[9] though in cases that were not pulmonary. In one case, in a male aged 54, the diagnosis of tuberculous cystitis, with probable latent (healed) lung lesion, was confirmed by the reactions to tuberculin. The first dose of ·004 grammes was given in February, and by various degrees of increase and intervals of administration doses of 80 milligrammes were injected. The reactions had been positive, but more general than febrile. By the time 80 milligrammes doses were reached he had gained in weight, spirits and looks, and the bacilli disappeared from the urine. By May 21st he had improved immensely.

A case of tuberculous disease of the right hip, rapid pulse, loss of weight, &c., a diagnostic dose of ·001 and ·0015 grammes gave positive reactions. This was in August, 1894. By April 23rd, 1895, having been receiving ·120 gramme injections every ten days, she had gained 21 lbs. weight, discarded her crutch and her general improvement was decided.

Another very similar case is reported. The author is of opinion that in the face of such evidence as this (and with proper discrimination the use of tuberculin in pulmonary tuberculosis has a good defence) he is justified in saying that one such case as the second here given is worthy of more credence than a hundred failures by others who, as likely as not, have not appreciated the remedy or the conditions under which it should be used. Of 130 tuberculous patients treated with tuberculin, all but one of 33 without softening (excluding a few of whom traces have been lost) are known to be living, and 45 out of 97 who had reached or passed the stage of softening in lung tissue, are also known to be living, of whom 27 are favourable results in single or double cavity cases.

Another important case in point is furnished by Adami,[10] who relates the history and post-mortem appearances in a patient who came under Koch's treatment in 1890 suffering from hæmoptysis, cough, night sweats, and progressive emaciation. After a year's treatment by inoculation his health appeared to be entirely restored. In January, 1895, the old symptoms returned; in April he died of hæmoptysis. The post-mortem showed that the second attack was not the result of fresh infection, but of the recrudescence of the old process starting from the incompletely-healed diseased foci. Their nature indicated either relative attenuation of the bacilli, or increased resistance on the part of the tissues. In any case, a year's treatment by Koch's method had succeeded in arresting for four years an active and extensive tuberculous process in both lungs.

**Bromoform.**—This is advocated by K. Stepp[11] in the treatment of pulmonary tuberculosis, as well as in fibrinous pneumonia and asthma of emphysematous subjects, and the broncho-pneumonia of children. In such cases the favourable effects are accounted for by the fact that the remedy, taken internally, is eliminated from the system by the respiratory organs, and is thus enabled to exercise a direct beneficial effect on the affected parts. It is not suited for the ulcerative stage of pulmonary tuberculosis, but, on the other hand, in apyretic cases, without signs of breaking down of the lungs, but nevertheless greatly debilitated and freely expectorating, Stepp has found the daily administration of two to three grammes of bromoform result in greatly reduced expectoration and improvement in general condition. The ingestion of the remedy should be continued for weeks without interruption.

**Ichthyol.**—Scarpa[12] gives ichthyol with very encouraging results. He prescribes twenty drops daily of a mixture containing one part of sulpho-ichthyolate of ammonium, two parts of water, glycerine or alcohol, and a few drops of oil of peppermint. The dose is increased by ten drops every three or four days. The daily dose is poured into 300 to 500 grammes of water, of which the patient takes a mouthful from time to time.

**Climatic Treatment.**—There are few subjects presenting more genuine difficulty than that of indicating when we are justified in sending phthisical cases away from home. Samuel West,[13] writing on the sending of these cases abroad, considers those individual or personal factors in the problem which constitute its real difficulty, and upon the due appreciation of which the value of the advice given depends. He very cogently remarks that there are several questions to be answered before the right conclusion as to what course to advise can be arrived at. 1. Is the patient in such a condition as to be likely to derive permanent benefit from a better climate than he has at home? 2. If he is fit to travel, how long a journey can he undertake, and what kind of travelling will be the best? 3. What are his means (for foreign residence is largely a question of money)? 4. How long can he stay away from home? 5. Who can go with him? 6. What is the patient's general character, and how will it be affected by living in a foreign country, with house, customs, and food all strange to him? 9. How can he occupy his time or amuse himself? 10. What effect will separation from home and family have upon him? 11. As regards the place, we must consider (*a*) how far it will meet the various requirements already specified; (*b*) whether the patient is in a condition to derive full benefit from the advantages it offers; (*c*) the accommodation the place affords for an invalid in respect of housing, food, and general comforts; and (*d*) its accessibility, not only in respect of the journey out for the patient, but for the journey home should the patient desire to return, and especially wlth reference to the coming and going of friends—visits which go so far to make absence from home tolerable.

All these questions must be considered if wise advice is to be given. The most disastrous mistakes are often made by sending patients abroad without careful thought—disastrous alike to the patient, to the family, and to the practitioner's own reputation. The light and flippant way in which patients are sometimes advised to go abroad, with little or no regard to the special circumstances of each case, is often cruel in its results, and cannot be too strongly reprobated.

It is useless to send phthisical patients away for only a month or two. They should leave in October, and not return until May or June. Of course, they need not stay in the same place all the time. They may, for instance, spend the autumn in Geneva or at the Italian lakes, and make their way to the Riviera as winter sets in, going up, perhaps, to the mountains as the heat of spring and summer comes on. At any rate, the whole winter must be spent away, and if the first proves a success, possibly a second and a third. All this means money, and on this score the mind must be easy if the climate is to do good. Many other practical points are dealt with by the author which, though obvious enough, one would think, require to be emphasised, inasmuch as in practice they are so very generally ignored.

Egypt has of late years sprung into notoriety as a health resort, and its climatology has been the subject of several articles recently. Watson Williams[14] says that the climate of Egypt during the season from November to March is much the same as a moderately hot English summer, with perpetual sunshine during the day and cool evenings, but with this difference, that after the subsidence of the Nile the remarkable dryness of the atmosphere renders the air bracing and tonic, and prevents the sense of oppression one so often experiences in the moist heat of an English summer.

The following table, compiled by Dr. F. W. Sandwith[15] from the records of the five years 1884-88, the monthly bulletins being printed by the Khedivial Observatory in the Abbassiyeh suburb of Cairo, shows at a glance the mean temperature in this region, the dryness of the atmosphere, and the almost complete absence of rain and stormy wind:—

| | Barometer. | Thermometer Fahr. | | | Humidity. | | Rain in inches. | Clouds 0—10. | Wind. | |
|---|---|---|---|---|---|---|---|---|---|---|
| | | Mean of maxima. | Mean of minima. | Mean of means. | Relative. | Absolute. | | | Direction. | Force in miles. |
| January .. | 29.98 | 61.4 | 46.6 | 53.6 | 69.7 | .28 | .19 | 4.1 | S.W. | 2.2 |
| February ... | 29.95 | 65.3 | 48.8 | 57 | 66.2 | .29 | .24 | 4 2 | N. | 1.4 |
| March .. | 29.91 | 73.2 | 53 | 62.8 | 56.2 | .29 | .03 | 3.4 | N. | 2.5 |
| April ... ... | 29.82 | 81.2 | 59.9 | 70.4 | 47.8 | .31 | .12 | 3.4 | N. | 2.6 |
| May ... ... | 29.84 | 86.8 | 63.4 | 75.2 | 48.4 | .38 | .22 | 2.3 | N. | 2.8 |
| June ... .. | 29.78 | 94.7 | 70.2 | 82.6 | 44 | .44 | .02 | 1 | N. | 3 |
| July ... ... | 29.7 | 93 | 72.2 | 83.8 | 49 | .5 | — | 1.2 | N. | 4.3 |
| August ... | 29.7 | 92·9 | 71.4 | 82.2 | 55.3 | .54 | — | 1 6 | N. | 4.1 |
| September.. | 29.83 | 87.5 | 68 | 77.8 | 62.1 | .54 | — | 1 8 | N. | 4 3 |
| October ... | 29.89 | 84 | 64.8 | 74.3 | 65.8 | .52 | — | 2 5 | N. | 3.2 |
| November... | 29.96 | 74.2 | 56.3 | 64.4 | 67.5 | .39 | .21 | 3 | N. | 2.1 |
| December.. | 29.99 | 67.7 | 50.4 | 58.4 | 69.6 | .33 | .19 | 3.7 | N. | 2.2 |
| Average... | 29.86 | 80.1 | 60.4 | 70.2 | 58.46 | .40 | 1.22 | 2.6 | N. | 2.9 |

But the remarkably even temperature of Cairo is perhaps, more fully realised when we remember that "freezing point seems never to be reached in Cairo, while the indoor temperature of rooms on the north side of the house kept properly shut during the hot hours need never during the year exceed 83 deg. Fahr., and the same rooms during the depth of winter, if properly opened to receive the warm outer air during the day, need never have a temperature below 52 deg. Fahr., without employing a fire. By living in sunny south rooms, or by employing artificial heat, the invalid can be certain all night in the winter of a temperature of 65 Fahr., or more" (Sandwith). We have seen that the annual rainfall of Cairo is a little over an inch, at Helouan and Gizeh it is even less, while at Luxor and Assouan a shower of rain has been known to occur once every three or four years.

He proceeds to prove that the annual rainfall at Cairo is only one inch per annum, and at Luxor and Assouan practically nothing, and thns is far less than at Hyères, Algiers, Denver, &c.

During September and October the Nile rises and floods the low-lying ground on either side of its banks, but by the end of November the water has subsided, leaving immense tracts of moist fertile land. At night the dew, which is always present in Lower Egypt, is especially heavy during and for a month after "high Nile," so that invalids should avoid going out at night till the middle of December.

Yet the most remarkable feature of the Egyptian climate is the peculiar dryness of the atmosphere. At Cairo the mean relative humidity is 58·46, while even during the seven winter months it averages only 63·25, and in the daytime it is much lower, often reaching 10·0. At Gizeh and Helouan the humidity is considerably lower than at Cairo, the difference being still more remarkable at Luxor and Assouan when compared with London, where the humidity averages about 92·0 in winter. The importance of these figures is beyond dispute.

In Cairo, as in Alexandria, the water is supplied by a company which filters its water through large sand and gravel beds, and with subsequent filtration the water is excellent.

Tuberculous disease is by no means rare amongst the Arab and Coptic races; while the Soudanese, from the warmer and drier south, are very subject to consumption, a large percentage of the deaths at the hospitals being due to pulmonary tuberculosis.

Malaria is not common, and except that it not infrequently recurs in those who have suffered from previous attacks, in India or elsewhere, Europeans rarely suffer. Typhus fever is fairly common amongst the natives, but in the better classes it is seldom seen; the latter, on the other hand, often suffer from enteric fever, which the natives rarely contract. Visitors are most subject to diarrhœa from careless exposure to night air, with insufficient abdominal clothing; while pleurisy and pneumonia sometimes result from chill from similar want of due care in the night air. He dwells especially on the advantages of Helouan as a health resort. It is fourteen miles by rail from Cairo, in the desert, and as about twelve trains run either way daily it is very accessible from the capital. Helouan is probably the oldest spa in existence, as there is evidence to show that its sulphurous springs were used in the days of the Pharaohs. The sulphur and saline springs are utilised now in the treatment of chronic rheumatism and various other affections, a very complete set of baths having been erected on the Aix-les-Bains plan. But for consumptives its climate is especially suited, inasmuch as the surrounding desert renders the atmosphere very dry and sterile.

